# Adipose tissue macrophages in remote modulation of hepatic glucose production

**DOI:** 10.3389/fimmu.2022.998947

**Published:** 2022-08-24

**Authors:** Yan Tao, Quanhong Jiang, Qun Wang

**Affiliations:** Key Laboratory of Infection and Immunity of Shandong Province, Department of Immunology, School of Basic Medical Sciences, Cheeloo College of Medicine, Shandong University, Jinan, China

**Keywords:** hepatic glucose production, adipose tissue, macrophage, liver, gluconeogenesis

## Abstract

Hepatic glucose production (HGP) is fine-regulated *via* glycogenolysis or gluconeogenesis to maintain physiological concentration of blood glucose during fasting-feeding cycle. Aberrant HGP leads to hyperglycemia in obesity-associated diabetes. Adipose tissue cooperates with the liver to regulate glycolipid metabolism. During these processes, adipose tissue macrophages (ATMs) change their profiles with various physio-pathological settings, producing diverse effects on HGP. Here, we briefly review the distinct phenotypes of ATMs under different nutrition states including feeding, fasting or overnutrition, and detail their effects on HGP. We discuss several pathways by which ATMs regulate hepatic gluconeogenesis or glycogenolysis, leading to favorable or unfavorable metabolic consequences. Furthermore, we summarize emerging therapeutic targets to correct metabolic disorders in morbid obesity or diabetes based on ATM-HGP axis. This review puts forward the importance and flexibility of ATMs in regulating HGP, proposing ATM-based HGP modulation as a potential therapeutic approach for obesity-associated metabolic dysfunction.

## Introduction

Glucose is an essential energy source for most tissue cells in mammals. The circulation levels of glucose are strictly controlled within a stable range in healthy individuals. Abnormally low level of blood glucose, known as hypoglycemia, produces deleterious effects on physiological functions of various tissues and organs. Severe hypoglycemia substantially reduces glucose supply to the brain, resulting in central nervous system damage with consequent seizures, coma and death. In contrast, chronic hyperglycemia, a state of long-term high blood glucose usually in diabetes mellitus, may lead to dysfunction and/or failure of various organs (1). Thus, glucose homeostasis is critical for physiological fasting-feeding cycle particularly in overnutrition state.

During the fed state, dietary intake supplies exogenous glucose to provide energy; while under fasting conditions, endogenous glucose production becomes an indispensable way to maintain blood glucose levels. The liver produces endogenous glucose that accounts for the largest amount of glucose output. As such, the capacity for hepatic glucose production (HGP) plays a crucial role in maintaining blood glucose during fasting. Contrarily, aberrant elevations of HGP lead to hyperglycemia and associated metabolic disorders, particularly in individuals with morbid obesity and associated diabetes mellitus (2, 3). Thus, HGP modulation is a pivotal approach for the treatment of obesity-associated diabetes.

Adipose tissue closely interacts with the liver to regulate energy metabolism. In the fed state, triglycerides synthesized in hepatocytes are packed into VLDL and delivered into adipose tissue for storage (4). In the fasted state, the lipolysis of adipose tissue supplies the liver with non-esterified fatty acids (NEFAs) and glycerol that serve as the precursors of HGP (4–6). Besides metabolic products, adipose tissue communicates with the liver by exchanging diverse information including adipokines and extracellular vesicles (EVs) (5, 7). During these interactions, macrophages exhibit high heterogeneity in dynamic adipose tissue niches, playing flexible roles in metabolic and immune processes. Accumulating studies are unraveling the involvement of adipose tissue macrophages (ATMs) in hepatic glycolipid metabolism. Here, we review their emerging roles in modulating HGP in the context of physiological fasting-feeding cycle as well as obesity or diabetes. Related ATM profiles and regulatory mechanisms for hepatic gluconeogenesis are discussed.

## HGP

Hepatocytes produce endogenous glucose relying on gluconeogenesis and glycogenolysis. The former generates glucose from non-carbohydrate substrates, while the latter produces glucose through glycogen breakdown. Both glycogenolysis and gluconeogenesis contribute to HGP during fasting period, whereas glycogenolysis only exists during early fasting stages due to limited glycogen storage (3, 4, 8, 9).

### Hepatic gluconeogenesis

Gluconeogenesis is a biological process of glucose generation from non-carbohydrate precursors such as glycerol, lactate, pyruvate and glucogenic amino acids (8, 10). As part of the reverse reaction of glycolysis, gluconeogenesis requires four critical enzymes to bypass the irreversible steps. Pyruvate carboxylase (PC) carboxylates pyruvate derived from glycolysis into oxaloacetate, which is decarboxylated by phosphoenolpyruvate carboxykinase (PEPCK) to form phosphoenolpyruvate. Fructose-1,6-bisphosphatase catalyzes fructose 1,6-bisphosphate into fructose 6-phosphate; while glucose 6-phosphatase (G6Pase) dephosphorylates glucose-6-phosphate (G6P) into glucose (8, 11, 12). Hepatic gluconeogenesis is strictly regulated by hormones in different nutrition states. These hormones regulate gluconeogenic gene expression by promoting or suppressing the binding of specific transcription factors with their promoters. To date, multiple transcription factors including CREB, PGC-1α, FOXO1 have been identified to promote PEPCK and/or G6Pase transactivation (13). In fasting conditions, glucagon secretion is increased whilst insulin levels decreased. Upon binding to their receptors on hepatocytes, glucagon activates cAMP-PKA signaling to mediate CREB activation, thereby promoting PEPCK and G6Pase transcriptions. CREB-mediated hepatic gluconeogenesis can be further enhanced by coactivator p300/CBP and CRTC2. In addition, CREB stimulates PGC-1α expression, another coactivator to cooperate with FOXO1 in promoting PEPCK and G6Pase transcriptions. During the fed state, insulin secretion is stimulated whilst glucagon production is inhibited. Insulin signaling mediates downstream AKT activation that phosphorylates FOXO1 for degradation, thereby reducing PEPCK and G6Pase transactivation (4, 9, 14–16). Fasting-induced glucocorticoid also contributes to hepatic gluconeogenesis through direct transactivation of PEPCK or G6Pase (17, 18). In case of hormone dysregulation, for instance, insulin resistance in human or animals with type 2 diabetes leads to the failure of FOXO1 degradation, causing hyperglycemia *via* abnormal gluconeogenesis (16, 19).

### Hepatic glycogenolysis

Glycogenolysis is a process of breaking down glycogen into glucose. Glycogen phosphorylase breaks down glycogen into glucos-1-phosphate, and the latter is converted into G6P by phosphoglucomutase, which is hydrolyzed into glucose by G6Pase. Glycogenolysis also indirectly contributes to hepatic gluconeogenesis by supplying glycolytic intermediates. A very recent study on mouse models provides evidence that glycogen in liver and muscle tissue supplies glycolytic intermediate lactic acid, hence contributing to gluconeogenesis even in the fed state (20).

## ATMs and their influences on HGP

There are two kinds of typical adipose tissue in mammals, white adipose tissue (WAT) responsible for energy storge and brown adipose tissue (BAT) responsible for energy dissipation (21). WAT has a close crosstalk with the liver to regulate metabolism and immune reactions. Under different pathophysiological conditions, heterogenous ATMs constitute special immune microenvironments, producing broad impacts on both adipose tissues and liver. Based on phenotypic characteristics, ATMs can be classified into M1 macrophages expressing high levels of proinflammatory cytokines like TNF-α, IL-1, IL-6, iNOS, and M2 macrophages with high levels of anti-inflammatory IL-10 and arginase 1. M2 macrophages are predominant in lean WAT to maintain immune hemostasis in healthy individuals, whereas M1 macrophages are accumulated in obese WAT and mediate adipose tissue inflammation and insulin resistance in metabolically unhealthy individuals. However, during metabolic switch from homeostasis to disorders, some phenotype markers may be overlapped between M1 and M2 macrophages and some potential markers have not been identified or included. There are still some difficulties to depict full and precise ATM profiles based on this simple classification, we therefore specify the phenotypic characteristics of ATMs related to HGP modulation here.

### Cytokines from ATMs on HGP Modulation

#### IL-10 in concert with insulin action: Physiological control of HGP

**Figure 1:**
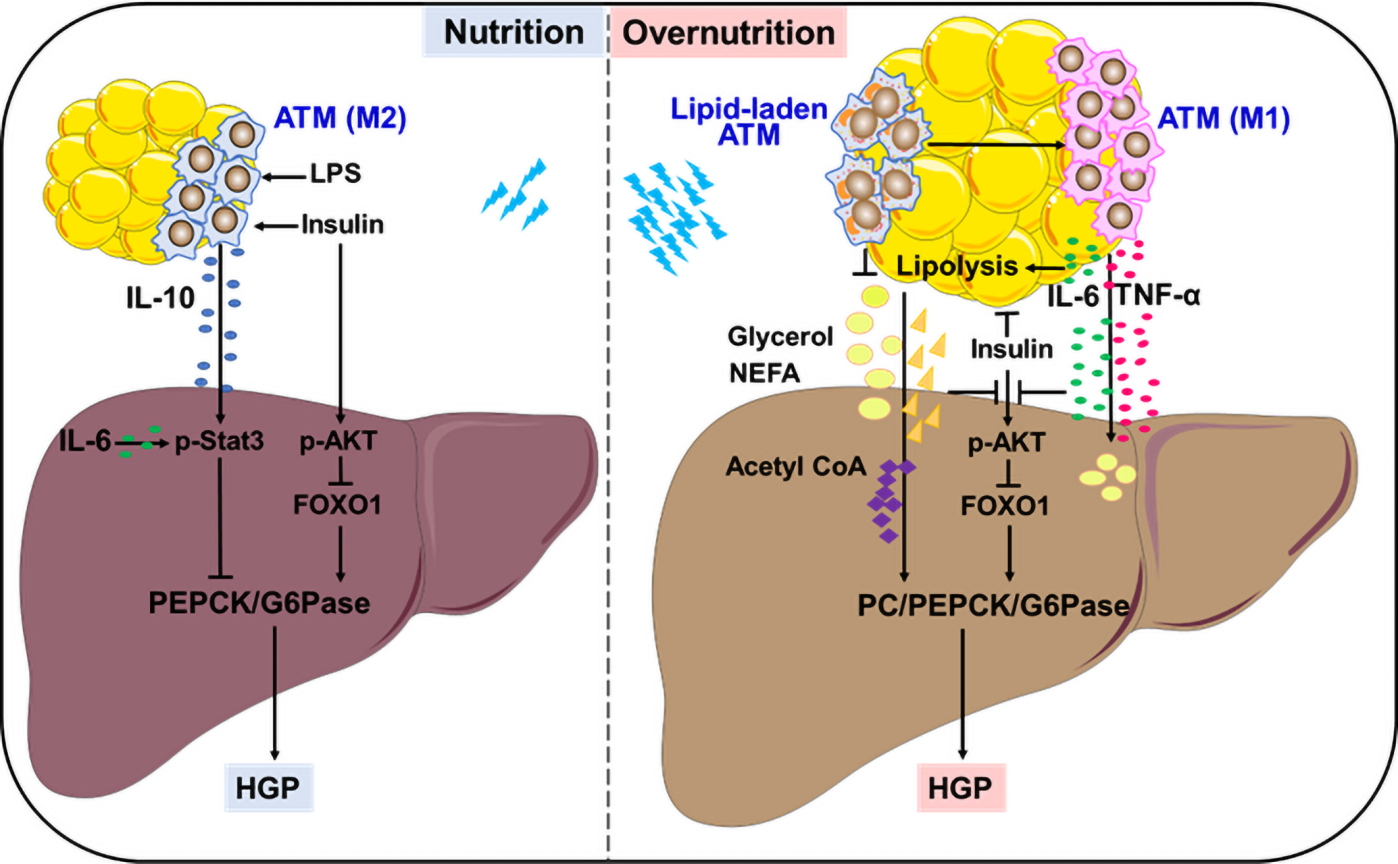
Remote modulation on HGP by ATMs. In the fed state, ATMs in response to insulin and LPS produce IL-10 to control gluconeogenesis-mediated HGP. In overnutrition state, ATMs secret large amounts of IL-6 and TNF-α, which link WAT inflammation with lipolysis, resulting in exacerbated HGP through interfering with insulin action or supplying precursors to stimulate aberrant gluconeogenesis. Lipid-associated ATMs play either protective or detrimental roles in controlling aberrant HGP.

Insulin is considered as the primary contributor to HGP suppression in the fed state. A recent study adds ATM-derived IL-10 to the list of HGP suppression, providing an elaborate explanation for feeding-induced HGP reduction. An earlier study showed that *in vitro* treatment with IL-10 plus IL-1β inhibited glucose production in primary rat hepatocytes through reducing PEPCK expression, but the underlying mechanisms remained unclear (22). Using different myeloid-specific gene knockout mouse models, Toda et al. demonstrated that macrophages, in response to postprandial signals LPS and insulin, produced IL-10 to suppress gluconeogenic gene expression and hepatocyte glucose production. This inhibitory effect was mediated by IL-10-stimulated Stat3 activation. Feeding induced IL-10 from ATMs, whereas obesity markedly decreased IL-10-producing macrophages in epidydimal WAT. Thus, ATM-derived IL-10 contributes to HGP suppression in cooperation with insulin in fed state (**Figure 1**). Upon obesity, ATMs with low IL-10 production failed to inhibit hepatic gluconeogenesis, resulting in high HGP and hyperglycemia. Importantly, IL-10 restoration successfully lowered the plasma glucose in obese mice, accompanied by the suppression of gluconeogenic genes in the liver (23, 24). Therefore, ATM-derived IL-10 may be a promising indicator to monitor HGP homeostasis during fasting-feeding cycle and a potential therapeutic target for obesity-induced metabolic dysfunction.

#### TNF-α in association with insulin resistance: Pathological stimulation of HGP

Insulin resistance is the major cause and typical feature of type 2 diabetes, which is closely associated with abnormal hepatocyte gluconeogenesis that contributes to enhanced HGP (16, 25, 26). An early study provided direct evidence that mice received constant infusion of TNF-α showed significant elevation in HGP together with decrease in insulin action and increase in glucagon secretion (27). Later, it was verified that TNF-α expression was significantly upregulated in WAT from rodents and human with obesity or diabetes, and closely associated with systemic insulin resistance. Importantly, TNF-α deficiency provided protection from obesity-induced insulin resistance in mice (28–30). ATMs are remarkably increased in obese WAT, which produce large amounts of TNF-α to drive insulin resistance and interfere with hepatic glycolipid metabolism (31, 32). TNF-α impairs insulin signaling by blocking tyrosine phosphorylation of insulin receptor substrate-1, the failure of AKT activation may facilitate HGP *via* glycogenolysis and gluconeogenesis. On the other hand, TNF-α may induce lipid overload in the liver, either from *de novo* lipogenesis through insulin-independent SREBP1 activation, or from excessive lipolysis of WAT elicited by insulin resistance (31, 33–35). Excessive lipolysis supplies lots of gluconeogenetic precursors like glycerol, NEFAs to promote aberrant HGP. Notably, more lipotoxic intermediators such as saturated palmitic acid and diacylglycerol are generated in these processes, which induce hepatic endoplasmic reticulum stress, inflammation and ensuing insulin resistance (5, 36–39). Therefore, TNF-α from obese ATMs may serve as a strong stimulator for abnormal HGP in obesity and associated diabetes mellitus (**Figure 1**).

#### IL-6 in cooperation with lipolysis: Indirect regulation of HGP

Besides TNF-α, IL-6 is another critical cytokine secreted from ATMs in obese condition. But differently, IL-6 regulates HGP in a more complex and flexible manner. To a large extent, the diversity of IL-6 modulation depends on its distinct target organs in different settings, which is more likely influenced by its sources and amounts. In fact, when directly acting on liver or primary hepatocytes, IL-6 at physiological concentrations elicited a suppression in glucagon-stimulated HGP and gluconeogenic genes (40, 41). In line with this, hepatic IL-6 that was induced by insulin action in the brain showed an inhibition on HGP through promoting Stat3 activation and reducing hepatocyte gluconeogenesis *via* PEPCK and G6pase suppression (42–44). Upon targeting adipocytes, IL-6 plays an indirect role in driving HGP process. In response to exercise or early fasting, skeletal muscle-derived IL-6 promotes lipolysis in WAT, thereby providing materials to support HGP (45–47). Of note, in case of morbid obesity, ATMs produce large amounts of IL-6 to stimulate excess WAT lipolysis, which abrogates insulin action on HGP suppression, resulting in hyperglycemia in rodents or adolescents (48, 49). Using *in vivo* metabolomics approach in combination with myeloid-specific JNK-deficient mice, and IL-6 neutralization or infusion treatment, Perry et al. provided evidence that insulin failed to suppress hepatic acetyl CoA, PC activity/flux and subsequent HGP in morbidly obese condition. During this process, IL-6 from ATMs played a key role in potentiating abnormal hepatic gluconeogenesis by increasing WAT lipolysis, leading to uncontrolled increases in hepatic acetyl CoA content and PC activity (**Figure 1**). These findings provide an alternative explanation for ATM-regulated, lipolysis-dependent HGP in diet-induced obesity, and propose a novel therapeutic target based on ATM-derived IL-6 (49). Moreover, the role of IL-6 in suppressing insulin signaling *via* SOCS3 induction in the liver should also be considered (48, 50, 51), which will not be detailed here.

#### ATMs on the bridge linking WAT inflammation with hepatic inflammation

As noted above, ATMs make pivotal contribution to hepatic insulin resistance through releasing proinflammatory cytokines. More interestingly, WAT inflammation also triggers hepatic inflammation, producing an overlapped interference on glycolipid homeostasis including HGP process. Through intraperitoneally transplanting visceral WAT from obese mice, Bijnen et al. established evident hepatic inflammation characterized by neutrophil and macrophage accumulation in receipt mice, which contributed to the development of non-alcoholic steatohepatitis (52). When ATMs were deleted from WAT prior to transplantation, this effect could be reversed. This study reveals the key position of ATMs in linking WAT inflammation and hepatic inflammation, in which CD11c^+^ ATMs that highly express neutrophil chemotactic genes are considered to recruit neutrophils followed by macrophages into the liver (52). In support of this, two earlier studies demonstrated an association of omental ATMs with hepatic inflammation and steatosis in insulin-resistant subjects, as well as the WAT inflammation prior to hepatic inflammation in mice during obesity development (53, 54).

### Lipid-laden ATMs on HGP Modulation

Besides the anti- or pro-inflammatory ATMs in lean or obese conditions, a peculiar population of lipid-laden ATMs intersecting metabolism and immunity is significantly upregulated by obesity in human and mouse WAT, particularly visceral WAT (55, 56). These lipid-laden ATMs not only showed an association with human body mass index, but also had an inhibitory effect on insulin signaling of fat explants from lean mice (56). Through analyzing the lipid profiles of ATMs in genetically obese (*ob/ob*) mice that were treated with or without rosiglitazone, Prieur et al. proposed that the cytotoxic lipid species including free cholesterol and saturated triglyceride could induce the switch of ATMs from M2 toward M1 phenotype, thereby resulting in obesity-induced inflammation and insulin resistance (55). In agreement with this, obese mice that received diet switching from high-fat diet to normal chow diet had a rapid normalization of metabolic parameters including reduced hepatic steatosis and gluconeogenesis-based HPG, accompanied by an obvious reduction in lipid-laden ATMs (57). These findings imply a close linkage of lipid-laden ATMs with liver metabolism. Notably, however, accumulating evidences demonstrated that some lipid-associated ATMs could improve glucose metabolic homeostasis by taking up and sequestering excessive lipids to avoid their damage on both adipose tissue and liver. Silence or deficiency of lipoprotein lipase or Trem2 associated with lipid metabolism abrogated the lipid uptake and storage in ATMs, resulting in insulin resistance and aggravating abnormal HGP *via* gluconeogenesis in obese mice (58, 59). As such, the lipid-associated ATMs may undergo dynamic changes with the development of obesity and have bidirectional regulation on HGP. It is very likely that ATMs assist in handling lipid during the metabolic adaptation to energy surplus, achieving a fine control of HGP through reducing lipid overload and toxicity on adipocytes and hepatocytes. By contrast, in case of lipid overload, toxic lipid species may stimulate ATMs to induce inflammation, insulin resistance and aberrant HPG, thereby producing deleterious effects on metabolic health (60, 61) (**Figure 1**). In agreement with this, an exosome-dependent transfer of neutral lipid from adipocytes to macrophages has recently been revealed (62, 63), further elucidation on lipid composition and amounts will help to elucidate their protective or detrimental effects on HGP modulation and consequent metabolic health.

### Potential ATM-based Modulators for HGP

Given the diversity of ATMs in different nutrition states, emerging ATM-associated modulators for HGP have been identified, and several potential targets are briefly summarized here.

#### Osteopontin (OPN)

OPN is a secreted matrix glycoprotein significantly upregulated in human and mouse adipose tissue upon obesity, and ATMs are its important source. OPN contributes to ATM accumulation *via* recruitment or proliferation in obese WAT, which is therefore closely associated with obesity-induced WAT inflammation, insulin resistance and HGP. Genetic deletion or antibody neutralization of OPN successfully alleviated ATM infiltration, WAT and hepatic inflammation in obese mice, thus improving insulin sensitivity accompanied by normalization of HGP markers. Of note, FOXO1 decrease mediated by AKT activation and Stat3 activation have been involved in HGP correction, confirming that OPN plays critical role in stimulating hepatic gluconeogenesis, though its direct or indirect effects remain to be carefully investigated (64–67). Interestingly, obese mice with selectively silenced OPN in epididymal ATMs showed an improvement in systemic glucose tolerance, further implying the contribution of ATM-derived OPN to the dysregulation of glucose metabolism. In addition, it should be noted that a population of recruited hepatic macrophages expressing OPN has recently been found to promote the development of non-alcohol fatty liver disease (68), which is warranted to be further studied regarding its regulation on HGP.

### Dipeptidyl peptidase 4 (DPP4)

DPP4 is a glycoprotein ubiquitously expressed by many cell types. Given its multiple roles in immune activation and metabolic influence, DPP4 has been considered as a potential therapeutic target for type 2 diabetes. DPP4 has a direct role in enzymatic cleavage of incretin hormone glucagon-like peptide-1, thereby interfering with normal glucose homeostasis. In addition, DPP4 is elevated in obese WAT where both adipocytes and ATMs are counted. Factors related to metabolic stress such as glucocorticoids and oxidized LDL have been reported to induce DPP4 upregulation on macrophages, which can stimulate macrophage inflammation and T cell activation, hence potentiating WAT inflammation and insulin resistance (69–72). As DPP4 can function in either soluble or membrane form, and hepatocytes also express DPP4, detailed functions of DPP4 including HGP modulation are yet to be determined.

### Retinol binding protein 4 (RBP4)

RBP4 is a serum retinol transporter secreted by the liver and fat. In obese mice, RBP4 stimulates ATM activation dependent on toll-like receptor 2/4, leading to production of proinflammatory cytokines like TNF-α, IL-12, IL-6, IL-1β, and subsequent T cell activation. This action plays a critical role in eliciting obesity-induced WAT inflammation and systemic insulin resistance (73–75). Interestingly, EVs from visceral WAT of obese mice were found to carry RBP4 that possessed the capacity to activate macrophages and elicit insulin resistance (76). More recently, a study on women with obesity linked RBP4 with HGP relying on its stimulation on adipocyte lipolysis. Direct stimulation on basal lipolysis as well as increased lipolysis by indirectly activating ATMs were both included (77). Therefore, RBP4 may act as another promising candidate for ATM-based HGP modulation, particularly in obesity-related glucose dysregulation.

## Concluding remarks and perspectives

Fine-regulation of HGP is critical to maintain blood glucose homeostasis in various nutrition states. HGP dysregulation contributes to hyperglycemia in obesity-associated diabetes. During fasting-feeding cycle or metabolic stress like overnutrition, ATMs change their cytokine or lipid profiles to remotely modulate HGP. Feeding induces IL-10 from ATMs, which controls HGP by inhibiting gluconeogenesis in concert with insulin action. While exacerbated HGP usually coincides with large amounts of IL-6 and TNF-α from ATMs, which link WAT inflammation with lipolysis, further interfering with insulin action or supplying precursors to stimulate gluconeogenesis. In response to overnutrition, lipid-associated ATMs play either protective or detrimental roles in controlling aberrant HGP (**Figure 1**). Therefore, ATM-based HGP modulation may be the promising strategies to treat obesity-associated diabetes.

Regarding ATM-based HGP modulation, more regulatory modes and targets remain to be unraveled, whilst some regulators of macrophages, such as a E3 ubiquitin ligase TRIM29 that inhibits macrophage activation and proinflammatory cytokine production (78), would be the promising candidates. Besides secretary molecules, EVs may be another indispensable remoter for fat-liver crosstalk. It is therefore interesting to explore whether and how EVs regulate the interactions between ATMs, adipocytes and hepatocytes, and their influences on HGP homeostasis. Upon challenged with energy surplus, WAT undergoes the process from metabolic adaptation to maladaptation, in which HGP-related ATM phenotypes and functions need to be carefully determined due to their complexity and flexibility. Given that gluconeogenesis is precisely regulated by circadian clocks, it is yet open but interesting to explore the changes of ATMs in this process. Furthermore, possible HGP regulation by BAT macrophages is also attractive, due to their involvement in energy metabolism (79, 80).

## Author contributions

YT and QJ drafted the manuscript. YT designed and created the figure. QW wrote and revised the manuscript, designed the figure. All authors approved the submitted version of the manuscript.

## Funding

This work is supported by the National Natural Science Foundation of China (81970733, 81770838, 81471065).

## Conflict of interest

The authors declare that the research was conducted in the absence of any commercial or financial relationships that could be construed as a potential conflict of interest.

## Publisher’s note

All claims expressed in this article are solely those of the authors and do not necessarily represent those of their affiliated organizations, or those of the publisher, the editors and the reviewers. Any product that may be evaluated in this article, or claim that may be made by its manufacturer, is not guaranteed or endorsed by the publisher.

## References

[1] American Diabetes A. Diagnosis and classification of diabetes mellitus. Diabetes Care (2009) 32(Suppl 1):S62–7. doi: 10.2337/dc13-S067. PMC2613584 19118289

[2] Gerich JE. Role of the kidney in normal glucose homeostasis and in the hyperglycaemia of diabetes mellitus: Therapeutic implications. Diabetes Med (2010) 27(2):136–42. doi: 10.1111/j.1464-5491.2009.02894.x PMC4232006 20546255

[3] Petersen MC Vatner DF Shulman GI. Regulation of hepatic glucose metabolism in health and disease. Nat Rev Endocrinol (2017) 13(10):572–87. doi: 10.1038/nrendo.2017.80 PMC5777172 28731034

[4] Rui L. Energy metabolism in the liver. Compr Physiol (2014) 4(1):177–97. doi: 10.1002/cphy.c130024 PMC4050641 24692138

[5] Targher G Corey KE Byrne CD Roden M. The complex link between NAFLD and type 2 diabetes mellitus - mechanisms and treatments. Nat Rev Gastroenterol Hepatol (2021) 18(9):599–612. doi: 10.1038/s41575-021-00448-y 33972770

[6] Kranendonk ME Visseren FL van Herwaarden JA Nolte-'t Hoen EN de Jager W Wauben MH. Effect of extracellular vesicles of human adipose tissue on insulin signaling in liver and muscle cells. Obes (Silver Spring) (2014) 22(10):2216–23. doi: 10.1002/oby.20847 25045057

[7] Fuchs A Samovski D Smith GI Cifarelli V Farabi SS Yoshino J. Associations among adipose tissue immunology, inflammation, exosomes and insulin sensitivity in people with obesity and nonalcoholic fatty liver disease. Gastroenterology (2021) 161(3):968–81.e12. doi: 10.1053/j.gastro.2021.05.008 PMC8900214 34004161

[8] Nuttall FQ Ngo A Gannon MC. Regulation of hepatic glucose production and the role of gluconeogenesis in humans: Is the rate of gluconeogenesis constant? Diabetes Metab Res Rev (2008) 24(6):438–58. doi: 10.1002/dmrr.863 18561209

[9] Cui A Ding D Li Y. Regulation of hepatic metabolism and cell growth by the atf/creb family of transcription factors. Diabetes (2021) 70(3):653–64. doi: 10.2337/dbi20-0006 PMC7897342 33608424

[10] McCommis KS Finck BN. Mitochondrial pyruvate transport: A historical perspective and future research directions. Biochem J (2015) 466(3):443–54. doi: 10.1042/BJ20141171 PMC4464838 25748677

[11] Beale EG Hammer RE Antoine B Forest C. Disregulated glyceroneogenesis: PCK1 as a candidate diabetes and obesity gene. Trends Endocrinol Metab (2004) 15(3):129–35. doi: 10.1016/j.tem.2004.02.006 15046742

[12] Hutton JC O’Brien RM. Glucose-6-phosphatase catalytic subunit gene family. J Biol Chem (2009) 284(43):29241–5. doi: 10.1074/jbc.R109.025544 PMC2785553 19700406

[13] Oh KJ Han HS Kim MJ Koo SH. CREB and FoxO1: Two transcription factors for the regulation of hepatic gluconeogenesis. BMB Rep (2013) 46(12):567–74. doi: 10.5483/BMBRep.2013.46.12.248 PMC4133859 24238363

[14] Nakae J Kitamura T Silver DL Accili D. The forkhead transcription factor Foxo1 (Fkhr) confers insulin sensitivity onto glucose-6-phosphatase expression. J Clin Invest (2001) 108(9):1359–67. doi: 10.1172/JCI200112876 PMC209440 11696581

[15] Lu M Wan M Leavens KF Chu Q Monks BF Fernandez S. Insulin regulates liver metabolism *in vivo* in the absence of hepatic akt and Foxo1. Nat Med (2012) 18(3):388–95. doi: 10.1038/nm.2686 PMC3296881 22344295

[16] Hatting M Tavares CDJ Sharabi K Rines AK Puigserver P. Insulin regulation of gluconeogenesis. Ann N Y Acad Sci (2018) 1411(1):21–35. doi: 10.1111/nyas.13435 PMC5927596 28868790

[17] Korenfeld N Finkel M Buchshtab N Bar-Shimon M Charni-Natan M Goldstein I. Fasting hormones synergistically induce amino acid catabolism genes to promote gluconeogenesis. Cell Mol Gastroenterol Hepatol (2021) 12(3):1021–36. doi: 10.1016/j.jcmgh.2021.04.017 PMC8346669 33957303

[18] Xu S Liu Y Hu R Wang M Stöhr O Xiong Y. TAZ inhibits glucocorticoid receptor and coordinates hepatic glucose homeostasis in normal physiological states. Elife (2021) 10:e57462. doi: 10.7554/eLife.57462. 34622775 PMC8555985

[19] Magnusson I Rothman DL Katz LD Shulman RG Shulman GI. Increased rate of gluconeogenesis in type-ii diabetes-mellitus - a c-13 nuclear-magnetic-resonance study. J Clin Invest (1992) 90(4):1323–7. doi: 10.1172/JCI115997 PMC443176 1401068

[20] TeSlaa T Bartman CR Jankowski CSR Zhang Z Xu X Xing X. The source of glycolytic intermediates in mammalian tissues. Cell Metab (2021) 33(2):367–78.e5. doi: 10.1016/j.cmet.2020.12.020 PMC8088818 33472024

[21] Peirce V Carobbio S Vidal-Puig A. The different shades of fat. Nature (2014) 510(7503):76–83. doi: 10.1038/nature13477 24899307

[22] Yerkovich ST Rigby PJ Fournier PA Olynyk JK Yeoh GC. Kupffer cell cytokines interleukin-1beta and interleukin-10 combine to inhibit phosphoenolpyruvate carboxykinase and gluconeogenesis in cultured hepatocytes. Int J Biochem Cell Biol (2004) 36(8):1462–72. doi: 10.1016/j.biocel2003.10.022. 15147725

[23] Toda G Soeda K Okazaki Y Kobayashi N Masuda Y Arakawa N. Insulin- and lipopolysaccharide-mediated signaling in adipose tissue macrophages regulates postprandial glycemia through akt-mtor activation. Mol Cell (2020) 79(1):43–53.e4. doi: 10.1016/j.molcel.2020.04.033 PMC11969070 32464093

[24] Sulen A Aouadi M. Fed macrophages hit the liver’s sweet spot with il-10. Mol Cell (2020) 79(1):1–3. doi: 10.1016/j.molcel.2020.06.016. 32619466

[25] Kubota N Kubota T Kajiwara E Iwamura T Kumagai H Watanabe t. Differential hepatic distribution of insulin receptor substrates causes selective insulin resistance in diabetes and obesity. Nat Commun (2016) 7:12977. doi: 10.1038/ncomms12977 27708333 PMC5059684

[26] Yan H Yang W Zhou F Li X Pan Q Shen Z. Estrogen improves insulin sensitivity and suppresses gluconeogenesis *via* the transcription factor foxo1. Diabetes (2019) 68(2):291–304. doi: 10.2337/db18-0638 PMC6341301 30487265

[27] Lang CH Dobrescu C Bagby GJ. Tumor necrosis factor impairs insulin action on peripheral glucose disposal and hepatic glucose output. Endocrinology (1992) 130(1):43–52. doi: 10.1210/endo.130.1.1727716 1727716

[28] Hotamisligil GS Shargill NS Spiegelman BM. Adipose expression of tumor necrosis factor-alpha: Direct role in obesity-linked insulin resistance. Science (1993) 259(5091):87–91. doi: 10.1126/science.7678183 7678183

[29] Hotamisligil GS Arner P Caro JF Atkinson RL Spiegelman BM. Increased adipose tissue expression of tumor necrosis factor-alpha in human obesity and insulin resistance. J Clin Invest (1995) 95(5):2409–15. doi: 10.1172/JCI117936 PMC295872 7738205

[30] Uysal KT Wiesbrock SM Marino MW Hotamisligil GS. Protection from obesity-induced insulin resistance in mice lacking TNF-alpha function. Nature (1997) 389(6651):610–4. doi: 10.1038/39335 9335502

[31] Shoelson SE Lee J Goldfine AB. Inflammation and insulin resistance. J Clin Invest (2006) 116(7):1793–801. doi: 10.1172/JCI29069 PMC1483173 16823477

[32] Weisberg SP McCann D Desai M Rosenbaum M Leibel RL Ferrante AW Jr. Obesity is associated with macrophage accumulation in adipose tissue. J Clin Invest (2003) 112(12):1796–808. doi: 10.1172/JCI200319246 PMC296995 14679176

[33] Yu YH Ginsberg HN. Adipocyte signaling and lipid homeostasis: sequelae of insulin-resistant adipose tissue. Circ Res (2005) 96(10):1042–52. doi: 10.1161/01.RES.0000165803.47776.38 15920027

[34] Leclercq IA Da Silva Morais A Schroyen B Van Hul N Geerts A. Insulin resistance in hepatocytes and sinusoidal liver cells: mechanisms and consequences. J Hepatol (2007) 47(1):142–56. doi: 10.1016/j.jhep.2007.04.002 17512085

[35] Rui L Aguirre V Kim JK Shulman GI Lee A Corbould A. Insulin/IGF-1 and TNF-alpha stimulate phosphorylation of IRS-1 at inhibitory Ser307 *via* distinct pathways. J Clin Invest (2001) 107(2):181–9. doi: 10.1172/JCI10934 PMC199174 11160134

[36] Stefan N Cusi K. A global view of the interplay between non-alcoholic fatty liver disease and diabetes. Lancet Diabetes Endocrinol (2022) 10(4):284–96. doi: 10.1016/S2213-8587(22)00003-1 35183303

[37] Stefan N. Causes, consequences, and treatment of metabolically unhealthy fat distribution. Lancet Diabetes Endocrinol (2020) 8(7):616–27. doi: 10.1016/S2213-8587(20)30110-8 32559477

[38] Steensels S Qiao J Zhang Y Maner-Smith KM Kika N Holman CD. Acyl-coenzyme a thioesterase 9 traffics mitochondrial short-chain fatty acids toward *de novo* lipogenesis and glucose production in the liver. Hepatology (2020) 72(3):857–72. doi: 10.1002/hep.31409 32498134

[39] Mashek DG. Hepatic lipid droplets: A balancing act between energy storage and metabolic dysfunction in NAFLD. Mol Metab (2021) 50:101115. doi: 10.1016/j.molmet.2020.101115 33186758 PMC8324678

[40] Dent JR Chowdhury MK Tchijov S Dulson D Smith G. Interleukin-6 is a negative regulator of hepatic glucose production in the isolated rat liver. Arch Physiol Biochem (2016) 122(2):103–9. doi: 10.3109/13813455.2016.1146773 26808480

[41] Christ B Yazici E Nath A. Phosphatidylinositol 3-kinase and protein kinase c contribute to the inhibition by interleukin 6 of phosphoenolpyruvate carboxykinase gene expression in cultured rat hepatocytes. Hepatology (2000) 31(2):461–8. doi: 10.1002/hep.510310228 10655271

[42] Ramadoss P Unger-Smith NE Lam FS Hollenberg AN. STAT3 targets the regulatory regions of gluconeogenic genes. in vivo Mol Endocrinol (2009) 23(6):827–37. doi: 10.1210/me.2008-0264 PMC5419286 19264844

[43] Inoue H Ogawa W Ozaki M Haga S Matsumoto M Furukawa K. Role of STAT-3 in regulation of hepatic gluconeogenic genes and carbohydrate metabolism. in vivo Nat Med (2004) 10(2):168–74. doi: 10.1038/nm980 14716305

[44] Inoue H Ogawa W Asakawa A Okamoto Y Nishizawa A Matsumoto M. Role of hepatic STAT3 in brain-insulin action on hepatic glucose production. Cell Metab (2006) 3(4):267–75. doi: 10.1016/j.cmet.2006.02.009 16581004

[45] Wueest S Item F Boyle CN Jirkof P Cesarovic N Ellingsgaard H. Interleukin-6 contributes to early fasting-induced free fatty acid mobilization in mice. Am J Physiol Regul Integr Comp Physiol (2014) 306(11):R861–7. doi: 10.1152/ajpregu.00533.2013 24694381

[46] Kistner TM Pedersen BK Lieberman DE. Interleukin 6 as an energy allocator in muscle tissue. Nat Metab (2022) 4(2):170–9. doi: 10.1038/s42255-022-00538-4 35210610

[47] Wedell-Neergaard AS Lang Lehrskov L Christensen RH Legaard DE Dorph E Larsen MK. Exercise-induced changes in visceral adipose tissue mass are regulated by il-6 signaling: a randomized controlled trial. Cell Metab (2019) 29(4):844–55.e3. doi: 10.1016/j.cmet.2018.12.007 30595477

[48] Hoene M Weigert C. The role of interleukin-6 in insulin resistance, body fat distribution and energy balance. Obes Rev (2008) 9(1):20–9.doi: 10.1111/j.1467-789X.2007.00410.x. 17956545

[49] Perry RJ Camporez JG Kursawe R Titchenell PM Zhang D. Hepatic acetyl CoA links adipose tissue inflammation to hepatic insulin resistance and type 2 diabetes. Cell (2015) 160(4):745–58. doi: 10.1016/j.cell.2015.01.012 PMC4498261 25662011

[50] Senn JJ Klover PJ Nowak JJ Zimmers TA Koniaris LG Furlanetto RW. Suppressor of cytokine signaling-3 (SOCS-3), a potential mediator of interleukin-6-dependent insulin resistance in hepatocytes. J Biol Chem (2003) 278(16):13740–6. doi: 10.1074/jbc.M210689200 12560330

[51] Kim JH Kim JE Liu HY Cao W Chen J. Regulation of interleukin-6-induced hepatic insulin resistance by mammalian target of rapamycin through the STAT3-SOCS3 pathway. J Biol Chem (2008) 283(2):708–15. doi: 10.1074/jbc.M708568200 17993646

[52] Bijnen M. Adipose tissue macrophages induce hepatic neutrophil recruitment and macrophage accumulation in mice. Gut (2018) 67(7):1317–27. doi: 10.1136/gutjnl-2016-313654 29074725

[53] van der Heijden RA Sheedfar F Morrison MC Hommelberg PP Kor D Kloosterhuis NJ. High-fat diet induced obesity primes inflammation in adipose tissue prior to liver in C57BL/6j mice. Aging (Albany NY) (2015) 7(4):256–68. doi: 10.18632/aging.100738 PMC4429090 25979814

[54] Tordjman J Poitou C Hugol D Bouillot JL Basdevant A Bedossa P. Association between omental adipose tissue macrophages and liver histopathology in morbid obesity: Influence of glycemic status. J Hepatol (2009) 51(2):354–62. doi: 10.1016/j.jhep.2009.02.031 19464069

[55] Prieur X Mok CY Velagapudi VR Núñez V Fuentes L Montaner D. Differential lipid partitioning between adipocytes and tissue macrophages modulates macrophage lipotoxicity and M2/M1 polarization in obese mice. Diabetes (2011) 60(3):797–809. doi: 10.2337/db10-0705 PMC3046840 21266330

[56] Shapiro H Pecht T Shaco-Levy R Harman-Boehm I Kirshtein B Kuperman Y. Adipose tissue foam cells are present in human obesity. J Clin Endocrinol Metab (2013) 98(3):1173–81. doi: 10.1210/jc.2012-2745 23372170

[57] Vatarescu M Bechor S Haim Y Pecht T Tarnovscki T Slutsky N. Adipose tissue supports normalization of macrophage and liver lipid handling in obesity reversal. J Endocrinol (2017) 233(3):293–305. doi: 10.1530/JOE-17-0007 PMC5457504 28360082

[58] Jaitin DA Adlung L Thaiss CA Weiner A Li B Descamps H. Lipid-associated macrophages control metabolic homeostasis in a trem2-dependent manner. Cell (2019) 178(3):686–98.e14. doi: 10.1016/j.cell.2019.05.054 PMC7068689 31257031

[59] Aouadi M Vangala P Yawe JC Tencerova M Nicoloro SM Cohen JL. Lipid storage by adipose tissue macrophages regulates systemic glucose tolerance. Am J Physiol Endocrinol Metab (2014) 307(4):E374–83. doi: 10.1152/ajpendo.00187.2014 PMC4137117 24986598

[60] Ertunc ME Hotamisligil GS. Lipid signaling and lipotoxicity in metaflammation: indications for metabolic disease pathogenesis and treatment. J Lipid Res (2016) 57(12):2099–114. doi: 10.1194/jlr.R066514 PMC5321214 27330055

[61] Caslin HL Bhanot M Bolus WR Hasty AH. Adipose tissue macrophages: Unique polarization and bioenergetics in obesity. Immunol Rev (2020) 295(1):101–13. doi: 10.1111/imr.12853 PMC8015437 32237081

[62] Flaherty SE Grijalva A Xu X Ables E Nomani A Ferrante AW Jr. A lipase-independent pathway of lipid release and immune modulation by adipocytes. Science (2019) 363(6430):989–93. doi: 10.1126/science.aaw2586 PMC6579605 30819964

[63] Antonyak MA Lukey MJ Cerione RA. Lipid-filled vesicles modulate macrophages. Science (2019) 363(6430):931–2. doi: 10.1126/science.aaw6765 30819952

[64] Nomiyama T Perez-Tilve D Ogawa D Gizard F Zhao Y Heywood EB. Osteopontin mediates obesity-induced adipose tissue macrophage infiltration and insulin resistance in mice. J Clin Invest (2007) 117(10):2877–88. doi: 10.1172/JCI31986 PMC1964510 17823662

[65] Kiefer FW Zeyda M Gollinger K Pfau B Neuhofer A Weichhart T. Neutralization of osteopontin inhibits obesity-induced inflammation and insulin resistance. Diabetes (2010) 59(4):935–46. doi: 10.2337/db09-0404 PMC2844841 20107108

[66] Kiefer FW Neschen S Pfau B Legerer B Neuhofer A Kahle M. Osteopontin deficiency protects against obesity-induced hepatic steatosis and attenuates glucose production in mice. Diabetologia (2011) 54(8):2132–42. doi: 10.1007/s00125-011-2170-0 PMC3131508 21562757

[67] Tardelli M Zeyda K Moreno-Viedma V Wanko B Grün NG Staffler G. Osteopontin is a key player for local adipose tissue macrophage proliferation in obesity. Mol Metab (2016) 5(11):1131–7. doi: 10.1016/j.molmet.2016.09.003 PMC5081407 27818939

[68] Remmerie A Martens L Thoné T Castoldi A Seurinck R Pavie B. Osteopontin expression identifies a subset of recruited macrophages distinct from kupffer cells in the fatty liver. Immunity (2020) 53(3):641–57.e14. doi: 10.1016/j.immuni.2020.08.004 PMC7501731 32888418

[69] Diaz-Jimenez D Petrillo MG Busada JT Hermoso MA Cidlowski JA. Glucocorticoids mobilize macrophages by transcriptionally up-regulating the exopeptidase DPP4. J Biol Chem (2020) 295(10):3213–27. doi: 10.1074/jbc.RA119.010894 PMC7062181 31988243

[70] Deacon CF. Physiology and pharmacology of DPP-4 in glucose homeostasis and the treatment of type 2 diabetes. Front Endocrinol (Lausanne) (2019) 10:80. doi: 10.3389/fendo.2019.00080 30828317 PMC6384237

[71] Rao X Zhao S Braunstein Z Mao H Razavi M Duan L. Oxidized LDL upregulates macrophage DPP4 expression *via* TLR4/TRIF/CD36 pathways. EBioMedicine (2019) 41:50–61. doi: 10.1016/j.ebiom.2019.01.065 PMC6441950 30738832

[72] Zillessen P Celner J Kretschmann A Pfeifer A Racké K Mayer P. Metabolic role of dipeptidyl peptidase 4 (DPP4) in primary human (pre)adipocytes. Sci Rep (2016) 6:23074. doi: 10.1038/srep23074 26983599 PMC4794806

[73] Moraes-Vieira PM Yore MM Sontheimer-Phelps A Castoldi A Norseen J Aryal P. Retinol binding protein 4 primes the NLRP3 inflammasome by signaling through toll-like receptors 2 and 4. Proc Natl Acad Sci USA (2020) 117(49):31309–18. doi: 10.1073/pnas.2013877117 PMC7733787 33214151

[74] Moraes-Vieira PM Yore MM Sontheimer-Phelps A Castoldi A Norseen J Aryal P. RBP4 activates antigen-presenting cells, leading to adipose tissue inflammation and systemic insulin resistance. Cell Metab (2014) 19(3):512–26. doi: 10.1016/j.cmet.2014.01.018 PMC4078000 24606904

[75] Moraes-Vieira PM Castoldi A Aryal P Wellenstein K Peroni OD Kahn BB. Antigen presentation and t-cell activation are critical for rbp4-induced insulin resistance. Diabetes (2016) 65(5):1317–27. doi: 10.2337/db15-1696 PMC4839203 26936962

[76] Deng ZB Poliakov A Hardy RW Clements R Liu C Liu Y. Adipose tissue exosome-like vesicles mediate activation of macrophage-induced insulin resistance. Diabetes (2009) 58(11):2498–505. doi: 10.2337/db09-0216 PMC2768161 19675137

[77] Kilicarslan M de Weijer BA Simonyté Sjödin K Aryal P Ter Horst KW Cakir H. RBP4 increases lipolysis in human adipocytes and is associated with increased lipolysis and hepatic insulin resistance in obese women. FASEB J (2020) 34(5):6099–110. doi: 10.1096/fj.201901979RR PMC7317205 32167208

[78] Xing J Weng L Yuan B Wang Z Jia L Jin R. Identification of a role for TRIM29 in the control of innate immunity in the respiratory tract. Nat Immunol (2016) 17(12):1373–80. doi: 10.1038/ni.3580 PMC5558830 27695001

[79] Wolf Y Boura-Halfon S Cortese N Haimon Z Sar Shalom H Kuperman Y. Brown-adipose-tissue macrophages control tissue innervation and homeostatic energy expenditure. Nat Immunol (2017) 18(6):665–74. doi: 10.1038/ni.3746 PMC5438596 28459435

[80] Rosina M Ceci V Turchi R Chuan L Borcherding N Sciarretta F. Ejection of damaged mitochondria and their removal by macrophages ensure efficient thermogenesis in brown adipose tissue. Cell Metab (2022) 34(4):533–48.e12. doi: 10.1016/j.cmet.2022.02.016 PMC9039922 35305295

